# Cow’s milk-based beverage consumption in 1- to 4-year-olds and allergic manifestations: an RCT

**DOI:** 10.1186/s12937-016-0138-0

**Published:** 2016-02-27

**Authors:** M. V. Pontes, T. C. M. Ribeiro, H. Ribeiro, A. P. de Mattos, I. R. Almeida, V. M. Leal, G. N. Cabral, S. Stolz, W. Zhuang, D. M. F. Scalabrin

**Affiliations:** 1 Federal University of Bahia, Salvador, Bahia Brazil; 2 Mead Johnson Pediatric Nutrition Institute, Evansville, IN USA

**Keywords:** DHA, Prebiotics, Yeast β-glucan, Allergic manifestations, Children

## Abstract

**Background:**

Nutrients such as docosahexaenoic acid (DHA), prebiotics and β-glucan have been associated with reduced incidence of respiratory illnesses and allergic manifestations (AM). Our objective was to assess if consumption of a cow’s milk-based beverage with these and other nutrients supports respiratory, gastrointestinal, and skin health in otherwise well-nourished, healthy children.

**Methods:**

In this double-blind, randomized, controlled trial, healthy children (1–4 years of age) from two daycare centers in Brazil were fed three servings/day of a cow’s milk-based beverage (CMBB; *n* = 125) containing DHA, the prebiotics polydextrose (PDX) and galactooligosaccharides (GOS), β-glucan, and other key nutrients, or a control cow’s milk-based beverage (control; *n* = 131) for up to 28 weeks. Occurrence of respiratory infections, diarrheal disease and AM was assessed by study pediatricians and the number of episodes were analyzed with the Cochran-Mantel-Haenszel test and the Andersen-Gill model.

**Results:**

The CMBB group had fewer episodes of AM, which included allergic rhinitis or conjunctivitis, wheezing, allergic cough, eczema and urticaria, compared to the control group (*p* = 0.021). The hazard ratio for increased number of episodes of AM was lower in the CMBB group compared to control (HR, 0.64; 95 % CI 0.47–0.89; *p* = 0.007). There was no difference in the incidence of respiratory infections and diarrheal disease between groups.

**Conclusion:**

A cow’s milk-based beverage containing DHA, PDX/GOS, and yeast β-glucan, and supplemented with micronutrients, including zinc, vitamin A and iron, when consumed 3 times/day for 28 weeks by healthy 1- to 4-year-old children was associated with fewer episodes of allergic manifestations in the skin and the respiratory tract.

**Trial registration:**

registration number: NCT01431469

## Background

Two leading causes of morbidity and mortality among children younger than 5 years of age are respiratory infections and diarrheal disease [1], which can be interrelated, as diarrhea increases the risk of lower respiratory infections [1, 2]. The global rise of allergic diseases, including asthma, atopic dermatitis and allergic rhinitis, is another cause of concern in children, with a significant impact on quality of life [3–5]. Nutrition-related factors are responsible for 11 % of the total global disease burden in children younger than 5 years [6]. Children are particularly vulnerable to diet inadequacies, which can compromise various mechanisms of immune function, thereby increasing risk of infections [7, 8] and also of allergic diseases, since oral tolerance to antigens may be impaired [9]. A recent study in 2- to 6-year-old daycare children from all regions in Brazil showed that even those of the highest socioeconomic levels had insufficient consumption of fiber and micronutrients, including calcium and vitamins D and E [10]. A systematic review concluded that apparently well-nourished children younger than 5 years from developed countries have diets that are inadequate in meeting the recommendations, placing them at nutritional risk [11]. Therefore, even diets of eutrophic children have room for improvement.

There is currently no agreement on the impact of a dietary improvement in children whose nutrient intake meets minimal requirements but may not be the most effective to promote improved health outcomes. Certain nutrients support the immune system, such as long-chain polyunsaturated fatty acids (LCPUFAs), especially omega-3 LCPUFAs, whose consumption has been associated with reduced allergic and/or respiratory illnesses in infants and children [12–16]. Likewise, prebiotic oligosaccharides support the immune system through stimulation of beneficial gut bacteria [17–20] and were associated with decreased respiratory infections and allergic diseases [21–24]. β-glucan, a polysaccharide derived from yeasts, fungi or bacteria, has also demonstrated immune-supporting properties [25, 26], with data showing protection against respiratory infections and allergy in adults and children [26–28]. In a recent randomized clinical trial in 3- to 4-year-old children attending daycare in China, we demonstrated that daily consumption of a cow’s-milk based beverage (CMBB) containing a combination of nutrients including docosahexaenoic acid (DHA), the prebiotics polydextrose (PDX) and galactooligosaccharides (GOS), and yeast β-glucan, and enriched with micronutrients such as vitamin A, zinc and iron, was associated with fewer acute respiratory infections (ARI) compared to cow’s milk [29].

Our objective in the current study was to evaluate if consumption of the nutritionally enriched CMBB used in the study in China had an effect on the incidence of ARI and diarrheal disease and secondarily on allergic manifestations (AM) in a distinct population of children attending daycare in Brazil. Typically, children in China start attending daycare when they are 3 year-old whereas in Brazil children usually start at daycare when they are 1 year old or earlier. Consequently, we wanted to assess in the current study if potential outcomes associated with consumption of the CMBB are influenced by age, ethnicity, climate and/or socioeconomic status. The implication would be to allow recommendation of the CMBB to children from different parts of the world with diverse ethnic and socioeconomic status.

## Methods

### Population

Children (1 up to 4 years of age) from 2 daycare centers in Salvador, Bahia, Brazil who had been consuming cow’s milk or cow’s milk-based beverage for at least 48 hours prior to randomization were eligible. Exclusion criteria were: > 50 % of total feedings consisting of breast milk; consumption of prebiotics or probiotics in the 15 days prior to randomization; diarrhea or ARI during the 48 h prior to randomization; a z-score of weight-for-height < −3; or any serious concurrent illness.

Eligible children were randomly assigned to one of two study products according to a computer-generated randomization sequence provided by the study sponsor (Mead Johnson Nutrition). The next sealed randomization envelope in sequence was opened to reveal the code of the product that the participant should receive. Product labels and randomization envelopes were created to prevent unblinding and the study products were similar in odor, color, and flavor (vanilla). Due to the broad variation of ages and to adjust for any potential impact of age on outcomes, participants were stratified at randomization into 12–24 months of age or 25–48 months of age. We estimated that children in these two age-range groups would be fairly homogenous in terms of potential outcomes related to the consumption of CMBB, including susceptibility to infections, diarrhea and AM.

The study was conducted from October 2011 to April 2012. The Federal University of Bahia Ethical Committee approved the protocol, and a parent/legal guardian provided signed informed consent prior to enrollment.

### Design

In this double-blind (participants and researchers), randomized, controlled, parallel-designed, prospective trial, children were fed an experimental CMBB, according to the CODEX definition for follow-up formula [30], with 25 mg of DHA, 1.2 g of a blend of PDX/GOS (1:1 ratio) and 8.7 mg of yeast β-glucan (Wellmune WGP®, Biothera, Eagan, Minnesota) per serving, or an isocaloric, non-supplemented cow’s milk-based beverage (control). Study products were given three times per day for 28 weeks, as a replacement for the usual breakfast, afternoon and dinner beverages. The breakfast and afternoon servings were offered by daycare attendants and the evening serving as well as weekend and holiday’s servings were offered at home by the caregiver. Each serving consisted of 40 g of powder mixed with 200 mL of water. The leftover of each serving was measured and recorded. See Table 1 for nutrient composition of study products.

**Table 1:** Nutrient composition of study products

Per 40 g serving of powder	Control	CMBB
Energy, kcal	180	180
Protein, g	7.3	7.3
Fat, g	6.6	6.6
- DHA, mg	--	25
Carbohydrate, g	23	23
- Dietary fiber, g (1:1 ratio PDX/GOS)	--	1.2
- Beta-1,3/1,6-glucans, mg	--	8.7
Vitamin A, IU	380	630
Vitamin D, IU	31	119
Vitamin E, IU	0.33	2.6
Vitamin K_1_, mcg	0.41	9.5
Thiamine, mcg	57	210
Riboflavin, mcg	520	490
Vitamin B_6_, mcg	42	183
Vitamin B_12_, mcg	0.72	0.72
Niacin, mcg	144	2200
Folic acid, mcg	7.8	31
Pantothenic acid, mcg	770	1160
Biotin, mcg	5.4	4.7
Vitamin C, mg	2.4	29
Choline, mg	28	44
Calcium, mg	280	290
Phosphorus, mg	200	187
Magnesium, mg	25	26
Sodium, mg	97	96
Potassium, mg	400	420
Chloride, mg	330	320
Iodine, mcg	13.4	15.2
Iron, mg	0.05	3.0
Zinc, mg	0.72	2.3
Manganese, mcg	5	19.2
Copper, mcg	4.8	82

-- Indicates that product did not contain the nutrient

### Outcomes

The primary outcome was incidence of ARI and/or diarrheal disease. ARI comprised upper respiratory infections, including common cold, pharyngitis, tonsillitis, otitis media, infectious sinusitis and rhinitis, and lower respiratory infections, including pneumonia, bronchiolitis and bronchitis [31]. Diarrheal disease was defined as ≥ 3 liquid or semi-liquid stools in 24 h with fever and/or vomiting and/or dehydration and compromised general status. Secondary outcomes included incidence of AM (allergic rhinitis or conjunctivitis, wheezing, allergic cough, eczema and urticaria) [31], incidence of all adverse events, growth, stool characteristics, fecal and serum immune markers, iron and zinc status and incidence of stool parasites.

In a *post hoc* analysis, we compared incidence of constipation during the study in the two study groups. Constipation was defined as presence of at least two of the following for at least two uninterrupted weeks: hard stools, difficulty or pain to defecate and a > 72-h interval without defecation.

All clinical outcomes were diagnosed by study pediatricians. Participants were evaluated by study pediatricians at the pediatric office in the daycare every time a health complaint was reported, either by parents/caregivers or daycare assistants. In addition, every 4 weeks participants were routinely assessed by study pediatricians at the time of anthropometric measurements. Weight and length/height measurements were obtained during randomization and every 4 weeks thereafter and converted into z-scores based on WHO growth standards [32]. Blood and stool samples were collected at baseline and end of study to assess peripheral blood cell count, serum ferritin and zinc, immune markers by ELISA (fecal secretory IgA and serum IL-10, TGF-β1, TGF-β2, IL-4 and IFN-ϒ) and stool parasites by direct microscopy. Laboratory analyses were conducted by R&D Systems, Minneapolis, MN, USA; Doctor’s Data, St. Charles, IL, USA; and the study site’s local lab.

### Sample size and statistics

A sample size of 125 completed per group was needed to achieve 90 % power, assuming a control group proportion of 0.5 and a test group proportion of 0.3 at an alpha level of 0.05. Frequencies of ARI, diarrheal disease and AM were compared using the Cochran-Mantel-Haenszel test stratified by age category and were further analyzed using the Andersen-Gill model with recurrent events modeled under the framework of the proportional hazards assumption. Fecal sIgA and serum TGF-β1 and TGF-β2 as well as their changes from baseline to end of study were compared using the van Elteren test stratified by age category. The Kruskal-Wallis test was used for all other serum immune markers, serum ferritin and zinc and peripheral blood counts, as well as changes in IL-10 and ferritin and zinc from baseline to end of study. Changes in peripheral blood counts were analyzed using ANCOVA, with baseline values as covariates. Stool frequency and consistency and weight- and length/height-for-age and weight-for-length/height z-scores were analyzed using repeated measures ANOVA.

## Results

### Study population and clinical outcomes

The study enrolled 256 children (control = 131; CMBB = 125); 2 discontinued in control and five in the CMBB group. Demographic and baseline characteristics (race, age, gender distribution and weight- and length/height-for-age and weight-for-length/height z-scores) were similar between groups. There were no growth differences between groups during the study. In both groups there was significant increase from baseline to end of study in weight- and length/height-for-age z-scores, as well as weight-for-length/height z-scores (females: 0.4 and 0.2 to 0.5 and 0.3; males: 0.4 and 0.4 to 0.5 and 0.5, in control and CMBB, respectively; *p* < 0.001). The average daily intake of study products was not significantly different between groups over the duration of the study (12–24 months of age: control 531 mL/day vs. CMBB 504 mL/day, *p* = 0.32; 25–48 months of age: control 547 mL/day vs. CMBB 498 mL/day, *p* = 0.06).

**Fig. 1:**
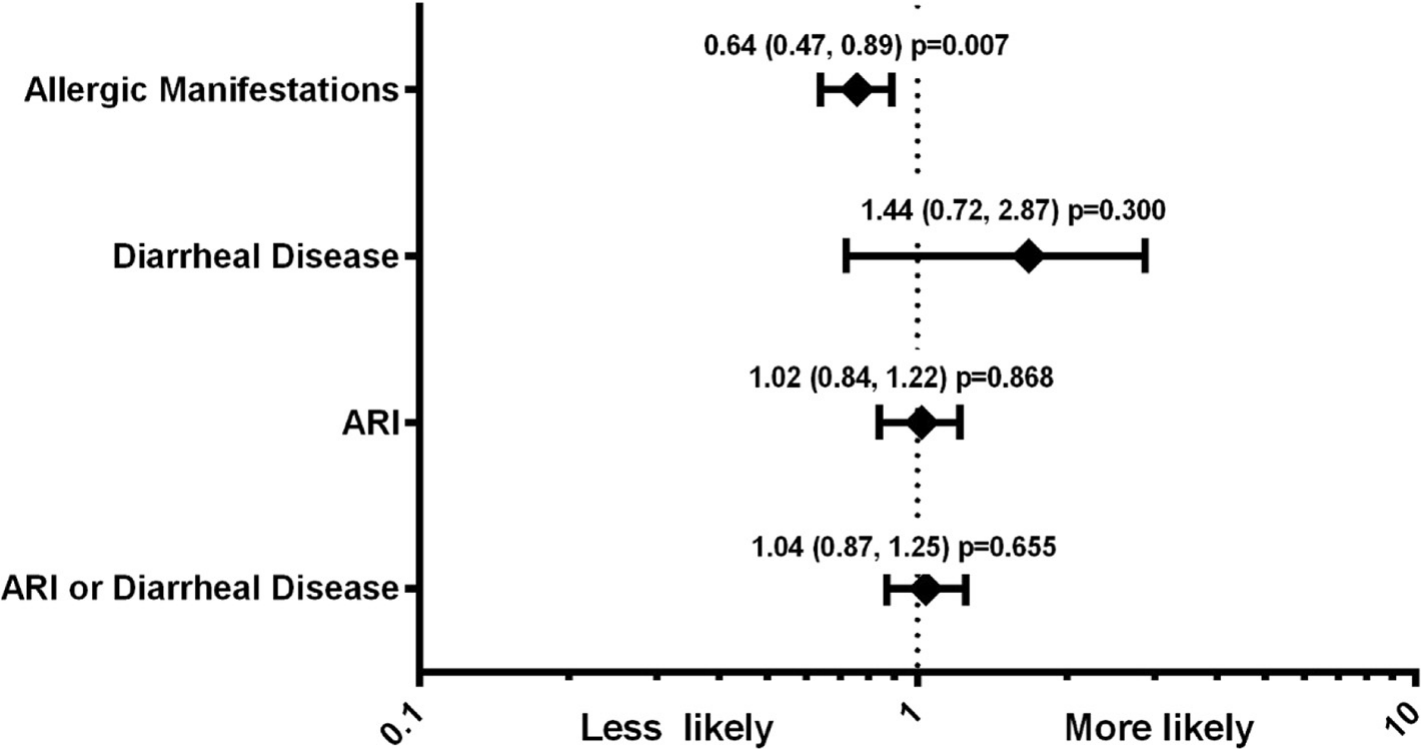
Hazard ratios (95 % CI) for increased number of episodes of illness using Andersen-Gill model adjusted for age category (12–24 or 25–48 months) and compared to control. ARI = acute respiratory infections

There was no difference in the incidence of ARI or diarrheal disease between groups. The CMBB group had fewer episodes of AM compared to control (Table 2). The hazard ratio for increased number of episodes of AM was lower in the CMBB group compared to control, with no difference for ARI or diarrheal disease (Fig. 1). There was no significant difference between groups in the hazard ratio of having at least one episode of ARI (0.93, 95 % CI 0.49, 1.79; *p* = 0.84), diarrheal disease (1.57, 95 % CI 0.71, 3.46; *p* = 0.26) or AM (0.61, 95 % CI 0.36, 1.04; *p* = 0.07). Among 99 types of adverse events reported and compared between groups, only occurrence of thrush was statistically different between groups (5 cases in CMBB vs. none in control; *p* = 0.03); 10 participants who experienced at least one serious adverse event were reported in the control vs. 2 in the CMBB group.

**Table 2:** Frequency of episodes of illness during the 28-week study period

	Number of episodes						*p*-value*
	None	1	2	3	4	>5	
Acute respiratory infections (ARI)							
Control; n (%)	25 (19)	40 (31)	28 (21)	21 (16)	10 (8)	7 (5)	0.938
CMBB; n (%)	25 (20)	40 (32)	24 (19)	16 (13)	14 (11)	6 (5)	
Diarrheal disease							
Control; n (%)	119 (91)	11 (8)	0 (0)	1 (1)	--	--	0.354
CMBB; n (%)	108 (86)	15 (12)	2 (2)	0 (0)	--	--	
Allergic manifestations (AM)							
Control; n (%)	71 (54)	30 (23)	19 (15)	10 (8)	1 (1)	--	0.021
CMBB; n (%)	81 (65)	28 (22)	12 (10)	3(2)	1 (1)	--	

*Statistical analysis used Cochran-Mantel-Haenszel test adjusted for age category

The CMBB group had softer stools compared with control in the first 3 months of the study (*p* ≤ 0.024). In the subgroup of children 12–24 months of age, 8 of 98 children (8 %) met the criteria for constipation. However, all were in the control group, and five of the eight (63 %) remained constipated at end of the study. In the subgroup of children 25–48 months of age, no significant difference was detected in the percentage of children who remained constipated at end of study (CMBB group 1/14; 7 % vs. control group 3/10; 30 %; *p* = 0.27).

### Blood and fecal outcomes

There were no differences between groups in any of the measured immune markers (Table 3). Additionally, no relevant differences were observed between groups for serum zinc and ferritin, hemoglobin, hematocrit, and red blood cells (Table 4); white blood cells and platelets (Table 5). According to WHO criteria (anemia: hemoglobin < 11 g/dL) [33], 18.04 % of the overall population was anemic at baseline and 13.33 % at end of study; 37.50 % was iron deficient at baseline and 45.83 % at end of study (iron deficiency: ferritin < 12 ng/mL) [33], with no differences between groups. Incidence of fecal parasites detected among the 17 assessed parasites is presented in Table 6.

**Table 3:** Comparison of immune markers between study groups

Variable^a^	Control median (IQR^b^)	CMBB median (IQR^b^)	*p*-value*
Fecal Secretory IgA, mg/dL			
Baseline	102 (12–226)	67 (7–228)	0.975
Week 28	45 (8–183)	32 (6–153)	0.452
Baseline to Week 28	−1 (−110–28)	−5 (−131–24)	0.664
IL-10, pg/mL			
Baseline	19.1 (11.9–27.5)	17.3 (12.0–27.6)	0.760
Week 28	13.9 (9.1–18.6)	13.6 (9.8–19.1)	0.771
Baseline to Week 28	−4.9 (−13.0–3.0)	−.39 (−12.0–0.0)	0.827
TGF-β1, pg/mL			
Baseline	20572 (15943–25641)	22386 (17161–29081)	0.109
Week 28	29133 (22432–35466)	29131 (22010–37532)	0.957
Baseline to Week 28	7863 (2569–14178)	6351 (−1346–12472)	0.132
TGF-β2, pg/mL^c^			
Baseline	344.1 (252.9–479.8)	326.0 (≤262.2–461.7)	0.801
Week 28	438.1 (296.7–600.2)	414.5 (≤331.0–586.3)	0.602
Baseline to Week 28	48.7 (0–123)	48.7 (0–125)	0.465
IL-4, pg/mL^d^			
Baseline	<1.6 (<1.6- < 1.6)	<1.6 (<1.6- < 1.6)	0.305
Week 28	<1.6 (<1.6- < 1.6)	<1.6 (<1.6- < 1.6)	0.305
IFN-ϒ, pg/mL^d^			
Baseline	<15.6 (<15.6- < 15.6)	<15.6 (<15.6- < 15.6)	0.368
Week 28	<15.6 (<15.6- < 15.6)	<15.6 (<15.6- < 15.6)	0.378

^a^All markers except fecal secretory IgA were measured in serum

^b^IQR = 25–75 % interquartile range

^c^28 % of samples were under the detection limit

^d^Changes from baseline to week 28 were not analyzed because most of the samples were under detection limit

*Van Elteren test stratified by age category was used for fecal secretory IgA, TGF-β1 and TGF-β2; Kruskal-Wallis test was used for all other immune markers

**Table 4:** Comparison of zinc, iron and red blood cell status between study groups

Variable	Control median (IQR^a^)	CMBB median (IQR^a^)	*p*-value*
Serum Zinc, μmol/L			
Baseline	21.4 (17.6–25.4)	21.8 (17.2–25.9)	0.793
Week 28	24.4 (20.5–29.5)	24.6 (20.2–28.2)	0.648
Baseline to Week 28	3.2 (−1.0–8.0)	3.3 (−2.0–8.0)	0.820
Serum Ferritin, ng/mL^b^			
Baseline	16.4 (<10–26.2)	14.8 (<10–21.8)	0.413
Week 28	13.5 (<10–19.6)	13.3 (<10–21.8)	0.863
Baseline to Week 28	−2.2 (−10.0–3.0)	0.0 (−7.0–6.0)	0.148
Hemoglobin, g/dL			
Baseline	11.8 (11.2–12.3)	11.7 (11.1–12.3)	0.432
Week 28	12.0 (11.4–12.6)	11.9 (11.4–12.5)	0.595
Baseline to Week 28^c^	0.26 (0.1)	0.30 (0.1)	0.561
Hematocrit, %			
Baseline	35.5 (34.0–37.1)	35.3 (33.8–37.2)	0.560
Week 28	35.1 (33.0–36.9)	34.9 (33.4–36.8)	0.736
Baseline to Week 28^c^	−0.41 (0.2)	0.32 (0.2)	0.747
Red Blood Cells, x10^9^/mL			
Baseline	4.6 (4.4–4.8)	4.6 (4.4–4.8)	0.678
Week 28	4.6 (4.3–4.7)	4.5 (4.2–4.8)	0.200
Baseline to Week 28^c^	−0.05 (0.0)	−0.12 (0.00)	0.024

^a^IQR = 25–75 % interquartile range

^b^26 % of the samples were at or under the detection limit

^c^Changes from baseline to study week 28 were analyzed using analysis of covariance (ANCOVA), with baseline values as covariate; the values listed are adjusted mean (SE)

*Kruskal-Wallis test was used for all variables except the ones described in footnote c

**Table 5:** Comparison of white blood cells and platelets between study groups

Variable	Control median (IQR^a^)	CMBB median (IQR^a^)	*p*-value*
White Blood Cells, x10^6^/mL			
Baseline	9.7 (7.5–11.4)	9.1 (7.3–11.6)	0.471
Week 28	8.3 (7.2–10.6)	8.5 (7.2–10.1)	0.710
Baseline to Week 28^b^	−0.95 (0.2)	−0.75 (0.2)	0.565
Neutrophils, %			
Baseline	41.0 (29.5–49.5)	36.4 (28.4–45.5)	0.069
Week 28	38.8 (28.9–47.2)	37.2 (29.9–45.3)	0.371
Baseline to Week 28^b^	0.25 (1.0)	−0.57 (1.1)	0.578
Lymphocytes, %			
Baseline	45.7 (35.0–55.7)	50.9 (40.2–58.0)	0.028
Week 28	47.1 (37.6–56.3)	48.3 (40.8–56.5)	0.230
Baseline to Week 28^b^	−0.26 (1.0)	0.99 (1.0)	0.390
Platelets, x10^6^/mL			
Baseline	341 (286–401)	345.5 (301.5–400.5)	0.760
Week 28	315 (266–369)	311 (263–367)	0.771
Baseline to Week 28^b^	−3.9 (0.77)	−3.7 (.82)	0.837

^a^IQR = 25–75 % interquartile range

^b^Changes from baseline to study week 28 were analyzed using analysis of covariance (ANCOVA), with baseline values as covariate; the values listed are adjusted mean (SE)

*Kruskal-Wallis test was used for baseline and study week 28 values of all variables in this table

**Table 6:** Incidence of fecal parasites at baseline and study week 28 in the overall population^a^

Type of parasite	12–24 months of age n (%)	25–48 months of age n (%)
*Giardia duodenalis*		
Baseline	21 (21.6)	38 (24.4)
Week 28	36 (37.9)	37 (25.7)
*Blastocystis hominis*		
Baseline	2 (2.1)	4 (2.6)
Week 28	6 (6.3)	7 (4.9)
*Endolimax nana*		
Baseline	0 (0)	9 (5.8)
Week 28	5 (5.3)	4 (2.8)
*Entamoeba coli*		
Baseline	0 (0)	7 (4.5)
Week 28	0 (0)	8 (5.6)
*Ascaris lumbricoides*		
Baseline	1 (1.0)	3 (1.9)
Week 28	0 (0)	4 (2.8)
*Trichuris trichiura*		
Baseline	0 (0)	3 (1.9)
Week 28	0 (0)	4 (2.8)
*Cryptosporidium sp.*		
Baseline	3 (3.1)	0 (0)
Week 28	0 (0)	0 (0)
*Entamoeba histolytica*		
Baseline	0 (0)	0 (0)
Week 28	0 (0)	1 (0.7)

^a^Participants from both study groups combined. Participants who were symptomatic as per physician’s evaluation received anti-parasite treatment during the study

## Discussion

In this randomized, double-blind, controlled study we demonstrated for the first time that healthy 1- to 4-year-old children who consumed a CMBB with DHA, PDX/GOS and yeast β-glucan for 28 weeks had fewer AM episodes compared to children who consumed an unfortified, cow’s milk-based beverage. No effect on ARI or diarrheal disease was associated with consumption of the CMBB.

These results are consistent with studies linking protection against AM with LCPUFAs [12, 34], prebiotics [21, 22, 35] and β-glucan [28, 36, 37]. Dietary LCPUFAs were associated with less atopic dermatitis and wheezing [12, 34], and amelioration of symptoms in asthmatic children [38]. Likewise, prebiotics have been associated with reduced incidence of atopic dermatitis, wheezing and allergic rhinitis [21, 35]. β-glucan was shown to alleviate symptoms of asthma in children when injected subcutaneously for 8 weeks [39] and symptoms of allergic rhinitis in adults [28, 36, 37]. In an RCT, adults with seasonal allergic rhinitis receiving β-glucan 250 mg/day orally for 4 weeks had reduced nasal and eye symptoms compared with a group who received placebo [28]. Additionally, vitamin D deficiency has been correlated with respiratory allergy [40], thus the addition of vitamin D to the CMBB could have contributed to our results. Future studies may identify the contribution of individual nutrients to the present findings.

LCPUFAs modulate some aspects of the innate and adaptive immune systems via different mechanisms, affecting cell membrane fluidity, membrane receptors and signaling pathways. DHA can prevent NF-κB activation with consequent decrease in production of IgE and pro-inflammatory cytokines that initiate and prolong allergic reactions [41]. DHA metabolites such as resolvins and protectins also act to limit inflammation [42]. Prebiotics, through stimulation of gut bacteria, may cause skewing of the perinatal allergy-prone Th2 milieu towards a balanced Th1 immune pathway [43]. β-glucan polysaccharides may also induce anti-allergic mechanisms [44]. In asthmatic children, β-glucan promoted an increase of IL-10 cytokine [39], which can inhibit Th2 mediators and allergic inflammation [45]. Interestingly, higher exposure to microbial components endotoxin and β-glucan was associated with decreased risk of sensitization to inhalant allergens, in accordance with the hygiene hypothesis [46].

Similar to the study in China [29], there was no effect on diarrheal disease in the current study. Additionally, we found no effect of CMBB on ARI in the Brazilian cohort, in contrast to the study in China in which the CMBB group had fewer episodes of ARI and just one case of AM in the whole study population [29]. Several factors could explain the different results. The diverse racial and genetic backgrounds of the two populations may have differently affected the impact of the various nutrients of the CMBB on the immune system. The International Study of Asthma and Allergies in Childhood (ISAAC) Phase Three trial reported low prevalence of asthma and wheezing in the Asia-Pacific region and high prevalence in Latin America [47] and Brazil is among the countries with the highest prevalence of asthma [48]. Repeated episodes of ARI were more frequent in the Brazil study, likely due to the younger age (1–4 years vs. 3–4 years in China) and predisposing conditions linked to lower socioeconomic level in the Brazil cohort.

The combined prevalence of *Ascaris lumbricoides* and *Trichuris trichiura* in our study (up to 5.6 % in the 2- to 4 year-olds at study week 28) is similar to the 5.4 % prevalence of these helminths reported in children attending daycare in Salvador, Brazil [49] and lower than the prevalence reported in daycare children in São Paulo (13 %) [50]. However, the prevalence of *Giardia duodenalis* in our study (up to 26 % in the 2- to 4-year-olds at study week 28) was higher than the prevalence of *Giardia* in those two studies [49, 50]. Intestinal parasites may have contributed to the high incidence of AM in our cohort, as suggested in a large survey in Brazilian children < 5 years [51], including *Giardia,* which was shown to be a risk factor for allergy [52].

Five children in the CMBB group, corresponding to 2 % of the overall population, were diagnosed with thrush; one of them had varicella and one had been on a recent course of antibiotic, both potential risk factors for thrush [53]. High rates of colonization with *Candida* are reported in healthy children, 12.5 % in 2 year-olds [54] and 45 % in 3 to 5 year-olds [55]. We found an incidence of thrush of 2.4 % in a previous study evaluating an experimental cow’s milk-based beverage in one of the daycare centers of the current study [56], corresponding to three cases in the control group and suggesting that thrush is not uncommon in that population of daycare children.

In the first 3 months of the study, the CMBB group had softer stools compared with control. We previously demonstrated that daycare children of a similar age receiving a CMBB with the same prebiotic blend used in this study had softer and more frequent stools [56]. There are very limited data on the role of prebiotics to alleviate constipation in young children [57]. In the current study, no conclusions can be made regarding the incidence of constipation in the 12- to 24- month age group, since no one in this group receiving the CMBB met the criteria for constipation in the *post hoc* analysis. In the small number of participants who met the criteria for constipation in the 25- to 48- month age group, fewer children receiving CMBB remained constipated at end of study than control, although the difference was not statistically significant.

An increase in weight and length/height z-scores from baseline to end of study was observed in both groups but there were no differences in growth between the two groups. According to standard criteria to diagnose malnutrition [58], none of the children were malnourished at enrollment. Inadequate adherence to dietary guidelines has been identified in apparently well-nourished children < 5 years of age [11]. Moreover, young children consuming unfortified cow’s milk were found to be at increased risk of insufficient intake of various nutrients, including iron and vitamin D, compared with those consuming a fortified CMBB [59, 60]. These data suggest that the use of a CMBB such as the one in this study may be justified to correct inadequate nutrient intake leading to hidden nutritional deficiencies that can impact a child’s health and development in the absence of an effect on growth. Iron deficiency, for instance, can lead to decreased cognitive function even in the absence of anemia [61]. Additionally, some nutrient deficiencies such as zinc deficiency impair normal appetite prompting a vicious circle; thus correction of the deficiency helps establish an adequate eating pattern [62].

There were no differences between groups in zinc and iron status at onset and end of study, with an incidence of anemia in the overall population of 18 and 13 %, at baseline and end of study, respectively. Reported prevalence of anemia in Brazil reached 47 % in children < 5 years of age, affecting all income strata but being higher in the poorest ones [63]. Participants in our study spent all weekdays at the daycare, receiving a high standard of dietary care, which likely contributed to the low incidence of anemia. Our findings are consistent with data showing a positive association between daycare attendance and hemoglobin level [64], which points to daycare attendance as protective against anemia.

The strengths of the current study include confirmation of diagnosis of ARI, diarrheal disease and AM as well as adverse events by experienced study pediatricians, close monitoring of the children at the daycare on a daily basis and meticulous assessment of intake of study formula. A weakness of the study is the inclusion of four immune active components and increased amount of minerals and vitamins in an experimental formula compared to a control formula without those improvements, which does not allow attributing the benefits of the experimental formula to individual components.

## Conclusion

In this study, regular consumption of a cow’s milk-based beverage containing DHA, PDX/GOS, and yeast β-glucan, and supplemented with micronutrients including zinc, vitamins A and D, and iron promoted improved immune outcomes, with fewer episodes of allergic manifestations in the skin and the respiratory tract in young children. These outcomes are highly relevant for a child’s overall health and physical, cognitive, psychological and social development and may contribute to the quality of life of the whole family, potentially decreasing school absenteeism, thus allowing less disruption on the lives of working parents. They may also have a direct economical impact linked to the cost of treating AM episodes, for parents and the health care system, and those costs likely surpass the cost of providing CMBB to the child.

Naturally occurring prebiotics, β-glucan and DHA are usually present in normal diet but their levels vary according to the quality of the diet and may not be sufficient to promote measurable health benefits. A CMBB such as the one used in this study may be of benefit in increasing the intake of such nutrients and correcting nutritional deficiencies that can affect immune function. We propose that a CMBB should be consumed in the context of a healthy, balanced diet. Since this is the first study showing a benefit of this CMBB on allergic manifestations in healthy young children, additional studies are warranted to confirm the current results and reinforce the recommendation of this formula to help reducing allergic manifestations.

### Ethics approval

The Research Ethics Committee of the University Hospital Complex Professor Edgard Santos, Federal University of Bahia, Brazil, and the National Research Ethics Committee (CONEP) approved this project on April 4, 2011 (NO. 76/2009).

## Abbreviations

AM: Allergic manifestation(s)
ANCOVA: Analysis of covariance
ANOVA: Analysis of variance
ARI: Acute respiratory infection(s)
CMBB: Cow’s milk-based beverage
DHA: Docosahexaenoic acid
GOS: Galactooligosaccharides
IFN: Interferon
Ig: Immunoglobulin
IL: Interleukin
LCPUFAs: Long-chain polyunsaturated fatty acids
NF: Nuclear factor
PDX: Polydextrose
RCT: Randomized controlled trial
TGF: Tumor growth factor
